# Clinical Efficacy and Safety of Low-Dose Pemafibrate in Patients With Severe Renal Impairment: A Retrospective Study

**DOI:** 10.7759/cureus.57777

**Published:** 2024-04-07

**Authors:** Hisato Shima, Manabu Tashiro, Tomoko Inoue, Kazuyoshi Okada, Takuya Okamoto, Seiichiro Wariishi, Toshio Doi, Jun Minakuchi

**Affiliations:** 1 Kidney Disease, Kawashima Hospital, Tokushima, JPN; 2 Nephrology and Hypertension, Kamei Hospital, Tokushima, JPN; 3 Laboratory, Kawashima Hospital, Tokushima, JPN; 4 Cardiovascular Surgery, Kawashima Hospital, Tokushima, JPN

**Keywords:** severe renal impairment, statins, chronic kidney disease (ckd), spparm alpha, hypertriglyceridemia, pemafibrate

## Abstract

Background: The management of hypertriglyceridemia in patients with chronic kidney disease (CKD) is important. Pemafibrate, a novel selective peroxisome proliferator-activated receptor-alpha modulator with less toxic effects on liver and kidney function than those of other fibrates, has recently been approved for the treatment of patients with an estimated glomerular filtration rate (eGFR) lower than 30 mL/min/1.73 m^2^. However, the efficacy and safety of pemafibrate in patients with severe renal impairment have not yet been established.

Methods: This single-center, retrospective observational study included 12 outpatients with CKD and hypertriglyceridemia, who were newly started on low-dose pemafibrate (0.1 mg/day) treatment between December 2021 and May 2023 and whose eGFRs were less than 30 mL/min/1.73 m^2^ at baseline. Blood samples were collected before and at 12 weeks after pemafibrate treatment.

Results: After 12 weeks of treatment, the serum triglyceride level was significantly decreased, whereas the high-density lipoprotein cholesterol level was significantly increased. The serum alanine aminotransferase, alkaline phosphatase, gamma-glutamyl transpeptidase, and uric acid levels were also significantly decreased, without worsening of the eGFR and serum creatinine levels. In the subgroup analysis, there were no significant differences in the changes in clinical parameters regardless of statin use and CKD stage at baseline.

Conclusions: Low-dose pemafibrate administration in patients with severe renal impairment resulted in significant improvements in triglyceride, high-density lipoprotein cholesterol, and serum uric acid levels, and liver function, without adverse events.

## Introduction

Chronic kidney disease (CKD) is a major public health problem and one of the strongest risk factors for cardiovascular disease (CVD) [1]. Patients with CKD typically have high levels of triglyceride (TG) and low levels of high-density lipoprotein cholesterol (HDL-C) [2]. Hypertriglyceridemia increases the risk of CVD and renal impairment, even though the level of low-density lipoprotein cholesterol (LDL-C) is well controlled [3-5]. Therefore, it is essential to manage hypertriglyceridemia in patients with CKD.

Pemafibrate, a newer-generation fibrate, was launched in Japan in 2018 prior to the rest of the world. As a novel selective peroxisome proliferator-activated receptor-alpha modulator (SPPARMα), it has shown a good safety profile and efficacy in the management of hypertriglyceridemia in patients with CKD [6]. According to the revised package insert of PARMODIA (Kowa, Nagoya, Japan), pemafibrate has recently been approved for the treatment of patients with an estimated glomerular filtration rate (eGFR) of less than 30 mL/min/1.73 m^2^. This has attracted much attention and may lead to increased use of the drug in patients with severe renal impairment. The revised package insert also included the following precautions: If the eGFR is less than 30 mL/min/1.73 m^2^, pemafibrate should be started at a low dose or administered at a longer interval. The maximum dose should be 0.2 mg/day. If its administration in combination with a statin is necessitated in patients with abnormal renal laboratory data, then pemafibrate should be started at a low dose. However, because the number of patients who had been administered the drug is small, the efficacy and safety of low-dose pemafibrate in individuals with severe renal impairment have not yet been established. Moreover, to the best of our knowledge, there are no reports on whether pemafibrate can be safely used in combination with a statin in such patients. Therefore, further clinical study is needed. This study aimed to assess the efficacy and safety of low-dose pemafibrate administration in patients with hypertriglyceridemia and severe renal impairment.

## Materials and methods

Study design

This single-center, retrospective observational study included a total of 13 outpatients with CKD and hypertriglyceridemia, who newly started low-dose pemafibrate (0.1 mg/day) treatment between December 2021 and May 2023 at Kawashima Hospital. The major inclusion criteria were as follows: patients aged ≧ 20 years, patients with fasting serum TG ≧ 150 mg/dL, patients treated with pemafibrate (0.1 mg/day), and patients with severe renal impairment (eGFR < 30 mL/min/1.73 m^2^) at the time of pemafibrate treatment initiation. The major exclusion criteria were patients receiving maintenance dialysis and patients with poor treatment adherence. The eGFR was estimated based on age, sex, and serum creatinine levels. We defined CKD as eGFR <60 mL/min/1.73 m^2^, diabetes mellitus as hemoglobin A1c ≧ 6.5% and/or taking anti-diabetic drugs, hypertension as systolic blood pressure ≧ 140 mmHg and/or diastolic blood pressure ≧ 90 mmHg and/or taking anti-hypertensive drugs, and hyperuricemia as serum uric acid (UA) ≧ 7.0 mg/dL and/or taking UA lowering drugs, respectively. The primary efficacy endpoint were the changes from baseline to week 12 in serum TG, HDL-C, and LDL-C levels. The primary safety endpoints included the incidence of rhabdomyolysis or the changes in liver and kidney functions. The secondary efficacy endpoints included changes or percent changes at week 12 compared to baseline, regarding parameters such as urinary protein, creatine kinase (CK), and UA. This study was approved by the Ethics Committee of Kawashima Hospital (Approval No. 1206). Because of the retrospective study design and anonymity of the patients studied, written informed consent was waived through the opt-out methodology. All clinical investigations were conducted according to the principles of the Declaration of Helsinki and Japanese ethical guidelines.

Data collection

Data on age, sex, past medical history, medical therapies affecting lipid metabolism, and laboratory findings both at the time of and 12 weeks after pemafibrate treatment initiation were collected by reviewing the medical records of the patients. Laboratory data included those of eGFR, serum creatinine, TG, HDL-C, LDL-C, urinary protein, aspartate aminotransferase (AST), alanine aminotransferase (ALT), alkaline phosphatase (ALP), gamma-glutamyl transpeptidase(γ-GTP), CK, and UA.

Statistical analysis

Data was expressed as the mean ± standard deviation (SD) or median (interquartile range) for continuous variables, as appropriate. Categorical variables are presented as numbers (percentages). Continuous variables were compared using Student’s t-test or the Mann-Whitney U test, as appropriate. All analyses were performed using JMP, version 17 (SAS Institute Inc., Cary, NC, USA). A p-value of less than 0.05 was considered statistically significant.

## Results

Baseline clinical and laboratory characteristics of the patients

One patient discontinued pemafibrate treatment owing to muscle symptoms, but no rhabdomyolysis was observed in any of the enrolled participants. Twelve patients were finally included in the analysis. The baseline clinical and laboratory characteristics of the patients are presented in Table 1.

**Table 1:** Baseline characteristics of the patients Values are presented as mean ± standard deviation or median (25%, 75%) for continuous variables, as appropriate. Categorical variables are presented as numbers (%). eGRF: estimated glomerular filtration rate; TG: triglyceride; LDL-C: LDL-cholesterol; HDL-C: HDL-cholesterol; AST: aspartate aminotransferase; ALT: alanine aminotransferase; ALP: alkaline phosphatase; γ-GTP: γ-glutamyl transpeptidase; CK: creatine kinase; UA: uric acid.

Variables	Value
Age (years)	65.1 ± 17.3
Female, n (%)	5 (41.7)
eGFR (mL/min/1.73m^2^)	18.1 ± 6.8
eGFR categories	
G4 (15≦ eGFR <30 mL/min/1.73m^2^), n (%)	7 (58.3)
G5 (eGFR <15 mL/min/1.73m^2^), n (%)	5 (41.7)
Serum creatinine (mg/dL)	2.82 (2.12, 4.09)
Proteinuria (g/gCr)	2.73 (1.34, 4.69)
Diabetes mellitus, n (%)	7 (58.3)
Hypertension, n (%)	9 (75.0)
Hyperuricemia, n (%)	11 (92.0)
Medical therapy	
Statin	6 (50.0)
Ezetimibe	2 (16.7)
TG (mg/dL)	338.5 (254.3, 754.3)
LDL-C (mg/dL)	100.0 (80.0, 121.3)
HDL-C (mg/dL)	43.8 ± 10.7
AST (U/L)	20.0 (18.0, 24.8)
ALT (U/L)	16.9 ± 4.8
ALP (U/L)	70.5 (59.8, 78.0)
γ-GTP (U/L)	28.0 (16.0, 42.0)
CK (U/L)	161.7 ± 107.7
UA (mg/dL)	7.4 ± 1.5

The mean age was 65.1 ± 17.3 years, and 41.7% were women. The mean eGFR was 18.1 ± 6.8 mL/min/1.73 m^2^. With regard to the eGFR, seven and five patients were in the G4 (15 ≦eGFR < 30 mL/min/1.73 m^2^) and G5 subgroups (eGFR < 15 mL/min/1.73 m^2^), respectively. Moreover, 58.3% of the participants had diabetes, 75.0% had hypertension, and 92.0% had hyperuricemia. At the time of pemafibrate treatment initiation, 50% of the patients were also on statins and 16.7% were on ezetimibe.

Comparisons between baseline and Week 12

**Figure 1:**
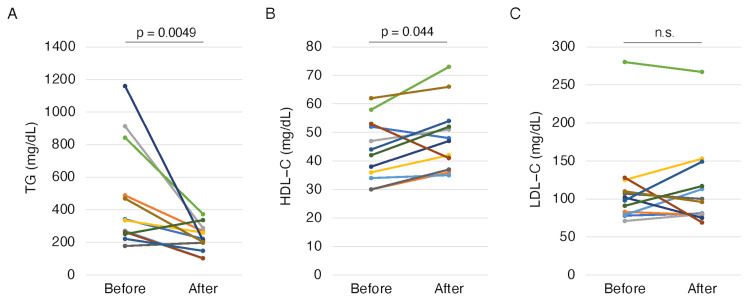
Changes in TG (A), HDL-C (B), and LDL-C (C) from before to after pemafibrate treatment. P-values were calculated using the paired t-test for parametric variables and Wilcoxon signed rank test for non-parametric variables. TG: triglyceride, HDL-C: high-density lipoprotein cholesterol, LDL-C: low-density lipoprotein cholesterol, n.s.: not significant.

Figure 1 shows the efficacy of the pemafibrate treatment. After 12 weeks of treatment, the serum TG levels had decreased significantly (p = 0.0049) (Figure 1A), whereas the HDL-C level had increased significantly (p = 0.044) (Figure 1B). By contrast, no significant difference in the LDL-C level was observed (Figure 1C).

**Figure 2:**
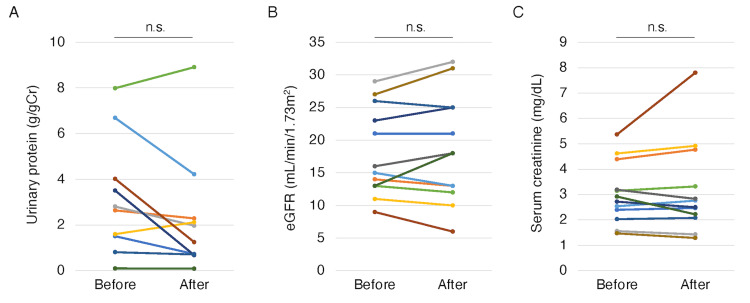
Changes in urinary protein (A), eGFR (B), and serum creatinine (C) from before to after pemafibrate treatment. P-values were calculated using the paired t-test for parametric variables and Wilcoxon signed-rank test for non-parametric variables. eGFR: estimated glomerular filtration rate, n.s.: not significant.

Figure 2 shows the effects of Pemafibrate on urinary protein levels and renal function. The urinary protein, eGFRs, and serum creatinine levels after the treatment were not significantly different from those before treatment (Figure 2A-2C).

**Figure 3:**
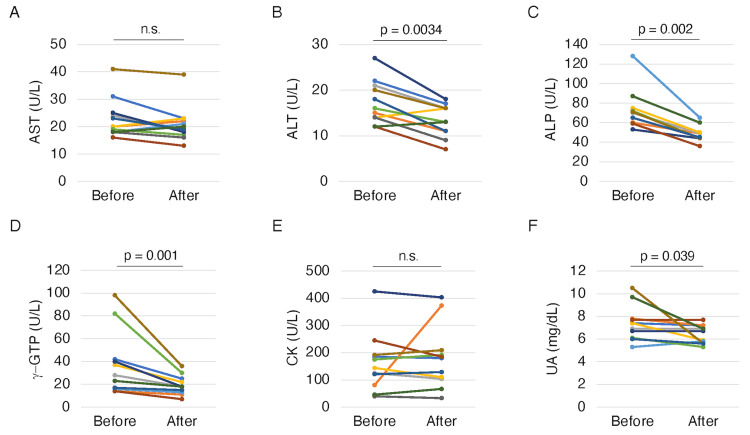
Changes in AST (A), ALT (B), ALP (C), γ-GTP (D), CK (E), and UA (F) from before to after pemafibrate treatment. P-values were calculated using the paired t-test for parametric variables and Wilcoxon signed-rank test for non-parametric variables. AST: aspartate aminotransferase, ALT: alanine aminotransferase, ALP: alkaline phosphatase, γ-GTP: γ-glutamyl transpeptidase, CK: creatine kinase, UA: uric acid, n.s.: not significant.

Figure 3 shows the effects of Pemafibrate on liver function and CK and UA levels. The serum ALT, ALP, γ-GTP, and UA levels were significantly decreased (p = 0.0034, 0.002, 0.001, and 0.039, respectively) (Figure 3B-3D, 3F), whereas the serum AST and CK levels did not change significantly (Figure 3A, 3E).

Changes in the clinical parameters from baseline to week 12 in patients who received pemafibrate without or with statin

We stratified the participants by the presence or absence of a statin at baseline. Table 2 shows the percent changes in lipid, CK, and UA levels and renal and liver functions from baseline to Week 12 in the two groups. There were no significant differences in these parameters between the groups.

**Table 2:** The percent change in lipid levels, renal and liver function, creatinine kinase, and uric acid from baseline to week 12 in patients who received pemafibrate without or with statins. Values are presented as median (interquartile range). The Mann-Whitney U test was used to compare differences between the two groups. A p-value of less than 0.05 was considered to be significant. eGFR: estimated glomerular filtration rate; TG: triglyceride; LDL-C: LDL-cholesterol; HDL-C: HDL-cholesterol; AST: aspartate aminotransferase; ALT: alanine aminotransferase; ALP: alkaline phosphatase; γ-GTP: γ-glutamyl transpeptidase; CK: creatine kinase; UA: uric acid.

Variables	Pemafibrate without statin (n=6)	Pemafibrate with statin (n=6)	p
Change in eGFR (%)	−5.8 (−10.2, 10.2)	5.2 (−13.7, 19.0)	0.47
Change in serum creatinine (%)	3.9 (−9.1, 7.0)	−2.7 (−14.5, 17.8)	0.81
Change in proteinuria (%)	−12.3 (−58.8, 22.0)	−29.9 (−60.3, −11.6)	0.53
Change in TG (%)	−56.3 (−66.8, −30.6)	−40.0 (−63.1, 17.0)	0.47
Change in LDL-C (%)	8.9 (−16.2, 45.3)	−0.49 (−16.4, 16.6)	0.58
Change in HDL-C (%)	19.7 (5.6, 24.2)	14.3 (−11.4, 23.5)	0.58
Change in AST (%)	−7.7 (−20.0, 15.4)	−14.9 (−22.1, 10.3)	0.47
Change in ALT (%)	−19.4 (−34.7, 9.8)	−25.2 (−37.2, −15.0)	0.34
Change in ALP (%)	−35.7 (−41.5, −27.3)	−30.5 (−37.0, −21.3)	0.59
Change in γ-GTP (%)	−47.8 (−63.3, −17.0)	−35.7 (−45.2, −21.6)	0.52
Change in CK (%)	6.6 (−14.4, 9.3)	−9.8 (−19.2, 124.4)	0.93
Change in UA (%)	−9.9 (−26.9, 2.4)	−2.7 (−18.3, 0)	0.78

Changes in the clinical parameters from baseline to Week 12 in patients with CKD G4 or G5

Finally, we stratified the clinical data by the eGFR subgroups G4 and G5 at baseline. There were no significant differences in the parameters between the two subgroups (Table 3).

**Table 3:** The percent change in lipid levels, renal and liver function, creatinine kinase, and uric acid from baseline to Week 12 in patients with CKD G4 or G5. Values are presented as median (interquartile range). The Mann-Whitney U test was used to compare the differences between the two groups. A p-value of less than 0.05 was considered to be significant. eGFR: estimated glomerular filtration rate; TG: triglyceride; LDL-C: LDL-cholesterol; HDL-C: HDL-cholesterol; AST: aspartate aminotransferase; ALT: alanine aminotransferase; ALP: alkaline phosphatase; γ-GTP: γ-glutamyl transpeptidase; CK: creatine kinase; UA: uric acid.

Variables	CKD G4 (n=7)	CKD G5 (n=5)	p
Change in eGFR (%)	8.7 (−3.8, 12.5)	−7.7 (−21.2, 15.7)	0.26
Change in serum creatinine (%)	−8.1 (−11.3, 2.9)	6.5 (−9.3, 27.0)	0.26
Change in proteinuria (%)	−36.9 (−66.1, −21.1)	−10.0 (−41.1, 22.0)	0.14
Change in TG (%)	−56.9 (−68.5, −33.3)	−44.9 (−58.5, 5.9)	0.33
Change in LDL-C (%)	3.8 (−12.7, 43.0)	−4.6 (−25.5, 25.5)	0.87
Change in HDL-C (%)	8.5 (2.9, 23.3)	20.0 (−3.0, 24.8)	0.52
Change in AST (%)	−17.4 (−25.8, −4.9)	10.0 (−14.6, 13.1)	0.19
Change in ALT (%)	−23.8 (−35.7, −20.0)	−18.8 (−34.2, 11.3)	0.46
Change in ALP (%)	−30.8 (−43.6, −23.5)	−33.3 (−38.9, −24.7)	0.68
Change in γ-GTP (%)	−38.1 (−57.1, −17.0)	−40.5 (−56.7, −21.6)	0.65
Change in CK (%)	−3.9 (−17.0, 7.2)	9.7 (−24.1, 203.1)	0.65
Change in UA (%)	−1.4 (−16.7, 2.4)	−13.1 (−24.6, −3.8)	0.23

## Discussion

To the best of our knowledge, this is the first study to evaluate the efficacy of low-dose pemafibrate administration in patients with severe renal impairment, half of whom were on statin therapy. Our results showed that low-dose pemafibrate significantly decreased the TG and increased the HDL-C levels without worsening renal function, even in patients with severe renal impairment. Pemafibrate also improved liver function and uric acid levels. Importantly, the results of this study suggest that low-dose pemafibrate administration is a promising therapeutic option for patients with severe renal impairment who were on statin therapy.

Hypertriglyceridemia in patients with CKD has been difficult to treat, because fibrates may induce rhabdomyolysis and deteriorate liver and kidney functions [7]. Aside from conventional fibrates, the SPPARMα drug pemafibrate can also maximize the on-target effects and minimize off-target effects such as rhabdomyolysis and liver and kidney impairment [8]. Additionally, pemafibrate is mainly metabolized in the liver and excreted into the bile, whereas conventional fibrates are mainly excreted from the kidney [9]. Although pemafibrate is usually prescribed at 0.1 mg twice daily (0.2 mg/day), the efficacy of low-dose administration (0.1 mg/day) has also been reported [10, 11]. The administration of fibrates in combination with statins was reported to increase the risk of rhabdomyolysis, particularly in patients with severe renal impairment [12]. In this study, we started with low-dose pemafibrate (0.1 mg/day), and one patient discontinued the treatment owing to muscle symptoms. However, no rhabdomyolysis was observed in any of the participants regardless of the use of statins, suggesting that low-dose pemafibrate administration can be safely used in combination with statins without worsening renal function, even in patients with severe renal impairment. Moreover, pemafibrate may be safely used regardless of eGFR categories (G4 and G5) at baseline.

Although fibrates have been reported to reduce proteinuria [7], pemafibrate did not have this effect in this study. Further studies with a larger number of patients and longer follow-up periods are needed. Fibrates such as fenofibrate can worsen liver function indicators such as ALT and γ-GTP [9]. However, the results of this study suggest that pemafibrate could improve these values. According to Honda et al. [13], pemafibrate improves the pathogenesis of non-alcoholic fatty liver disease (NAFLD)/non-alcoholic steatohepatitis (NASH) by modulating lipid turnover and energy metabolism in the liver. Pemafibrate may have similar mechanisms in its improvement of liver function in patients with dyslipidemia [13, 14]. Furthermore, because a strong association between NAFLD/NASH and CKD has been found [15], pemafibrate may ultimately be a promising treatment option for CKD [16]. In this study, many of the patients had hyperuricemia, and their serum UA levels were significantly decreased by pemafibrate. One possible reason for this is that pemafibrate decreases the overexpression of urate transporter 1 and glucose transporter 9 owing to insulin resistance and improves systemic insulin resistance [17]; however, the precise mechanisms remain to be elucidated.

This study has several limitations. First, the observation period was short. To evaluate the long-term efficacy and safety of pemafibrate, a longer period of drug administration is needed. Second, the number of patients was small. This might be because the package insert of pemafibrate has recently been revised and the efficacy and safety of low-dose pemafibrate in patients with severe renal impairment have not yet been evaluated. Third, this was a retrospective observational study without a control group. This study might be insufficient in these limitations but may have attracted much attention to physicians who prescribe pemafibrate to patients with severe renal impairment. Further prospective studies including a larger number of patients should be performed.

## Conclusions

Low-dose pemafibrate administration in patients with severe renal impairment resulted in significant improvements in the TG, HDL-C, and serum uric acid levels and liver function. It did not worsen renal function and no adverse events were observed, even in patients on statin therapy. Therefore, low-dose pemafibrate administration in combination with statins may be an option for treating patients with dyslipidemia and severe renal impairment.
